# Using a Social Robot to Evaluate Facial Expressions in the Wild

**DOI:** 10.3390/s20236716

**Published:** 2020-11-24

**Authors:** Silvia Ramis, Jose Maria Buades, Francisco J. Perales

**Affiliations:** Departament de Matemàtiques i Informàtica, Universitat Illes Balears, 07122 Palma de Mallorca, Spain; silvia.ramis@uib.es (S.R.); josemaria.buades@uib.es (J.M.B.)

**Keywords:** social robots, human-robot interaction, convolutional neural network (CNN), facial expression recognition, affective computing

## Abstract

In this work an affective computing approach is used to study the human-robot interaction using a social robot to validate facial expressions in the wild. Our global goal is to evaluate that a social robot can be used to interact in a convincing manner with human users to recognize their potential emotions through facial expressions, contextual cues and bio-signals. In particular, this work is focused on analyzing facial expression. A social robot is used to validate a pre-trained convolutional neural network (CNN) which recognizes facial expressions. Facial expression recognition plays an important role in recognizing and understanding human emotion by robots. Robots equipped with expression recognition capabilities can also be a useful tool to get feedback from the users. The designed experiment allows evaluating a trained neural network in facial expressions using a social robot in a real environment. In this paper a comparison between the CNN accuracy and human experts is performed, in addition to analyze the interaction, attention and difficulty to perform a particular expression by 29 non-expert users. In the experiment, the robot leads the users to perform different facial expressions in motivating and entertaining way. At the end of the experiment, the users are quizzed about their experience with the robot. Finally, a set of experts and the CNN classify the expressions. The obtained results allow affirming that the use of social robot is an adequate interaction paradigm for the evaluation on facial expression.

## 1. Introduction

Affective computing is the study and development of systems that can recognize, interpret, process, and simulate human affects. It is an interdisciplinary field spanning computer science, psychology, and cognitive science [1]. In the particular case, facial expression recognition plays an important role in human-robot interactions [2]. Intelligent robots must be able to recognize, interpret and respond effectively to social signals from a human. A robot that is able to interpret emotions will have an improved capacity to make decisions and help humans [3]. In this context we assume that a facial expression can be somewhat correlated with a set of equivalent emotions in some particular cases (amusement) and for specific ethnicity (Caucasian, Asian, etc.) [4,5].

Studies such as [6] have demonstrated that a robot can affect its social environment beyond the person who is interacting with it. For example, studies of robots used in autism therapy [7] show that robots can influence how children interact with others. For that reason, facial expression recognition is important to shape a good human-robot interaction and get a better user experience. Since social robots can simulate empathy and decide the best way to interact according to the facial expression of the user. Robots equipped with expression recognition capabilities can also be a useful tool to get feedback in videogames, for example, since they can assess the degree of satisfaction of the users. They can act as mediators, motivate the user and adapt the game according to the user’s facial expressions. 

On the other hand, many previous works have demonstrated that the use of robots in the field of rehabilitation has a considerable effect in the improvement of the patients [8,9,10,11]. There are several types of social robots in the current market [12], but we can highlight the robot NAO [13], which is a humanoid robot with friendly aspect and pleasant voice. This contributes to have a better user experience. Many papers have used the social robot NAO [13] in their experiments as in [14,15,16], where the social component of natural interaction is common to all the proposed applications, in addition to be a tool for motivation in rehabilitation sessions.

In this paper, we have created a multimodal interaction system using the social robot NAO, since one of the purposes of this work is to use this system as a tool for training the facial expressions, where the social robot acts as a supervisor of the user’s level of success regarding the facial expression performed. This system allows replicating and learning in a playful way seven facial expressions (happy, sadness, disgust, anger, surprise, fear and neutral). This kind of experiment also seeks to encourage attention and motivation of users, especially people with special needs, as for example children with autism. However, the system can be also used as a user-experience evaluation tool, where the robot is adapted according to the user’s expressions (positive feedback) or as a new capture method to get a new dataset on facial expressions “on the flight” through natural interaction with the game.

Therefore, the first step to perform this work has been to design and develop a serious game to be able to recognize facial expressions using a social robot. In this paper, two goals are set using a social robot:

(1) Evaluate a trained neural network in facial expressions using a social robot which permits to test the CNN in a real environment with a completely new set of users.
(2) Measure the attention and interaction of the participants with a social robot through a questionnaire at the end of the experiment.

The experiment consists in a serious game to evaluate the facial expression made by the user in front of social robot. The robot acts as if it were an evaluator of actors and actresses. Then the robot interacts with the person according to his or her facial expression. With each recognized expression, the robot responds with a positive phrase to encourage the user with the game. This experiment allowed the evaluation of a trained CNN which is used by a social robot that interacts with 29 non-expert participants. The interaction between the robot and the participant (dialogues and the fluidity of movements) is also evaluated, as well as the attention (level of user’s concentration) and the difficulty to express a facial expression through a final interview with each participant. Since the participants were non-experts in this field, some of them did not know how to express some facial expression. 

In the design of the facial expression recognition system we have used a trained network described in Section 4. This network has been trained with several standard frontal-face databases. A facial expression of the same person can appear differently depending on brightness, background and posture. The image quality, colour intensity, resolution are specifications that depend on the capture process and environment. These can affect the classification accuracy, especially in cross-dataset evaluation. This is when the training set and test set come from different databases. If the training set and test set come from the same database, the classification accuracy is more satisfactory [17,18,19,20,21] than if they come from different databases [22,23], where the classification results may decrease up to a 49%.

Therefore, when we use a social robot which recognizes facial expressions, how do we know how reliable it is? Generally, the facial expression databases are labeled, and we can test them, but the captured images by a social robot are not labeled. Therefore, the results obtained by the CNN were also compared with the ground truth provided by 10 experts (like in [24]) in facial expression recognition, in order to validate the system. We have considered as experts the 10 persons that ranked best in an initial test with 30 participants and which a hit rate of 100% was obtained.

Section 2 introduces the most relevant related literature. In Section 3, we explain the performed experiment. In Section 4, we explain the design and procedure in detail. Section 5 is devoted to analyzing the obtained results. The last section lists the conclusions, reviews the main contributions and proposes future lines of work.

## 2. Literature Review

Human-robot interaction (HRI) is a multidisciplinary field with contributions from human-computer interaction (HCI), artificial intelligence, robotics, natural language understanding, design and social sciences [25]. Within this field, a growing interest in incorporating facial expression recognition capabilities in social robots has emerged, since it plays an important role in the recognition and understanding of human expressions by robots [2]. A social robot that is able to recognize facial expressions and associate these expressions with a mood will able to improve in decision-making and help humans. These robots would promote more effective and attractive interactions with users and lead to better acceptance by users [26], since the humans prefer to interact with machines in the same way that they interact with other persons. These robots can be used as research platforms, toys, educational tools or as therapeutic aids [27]. An area of interest in social interaction is that of “robot as a persuasive machine” [28], that is, the robot can change the behaviour, feelings or attitudes of humans. An example would be to use the robot as a mediator in human-human interaction, as in the therapy of autism [29] or use the robot as a support to people with dementia [30]. In [30] proposed the integration of a lifestyle monitoring technology (passive infrared and door contact sensors) and social support robotics, providing people with dementia with relevant reminders such as having breakfast or going to bed. Another area is “the robot as an avatar” [31]. For example, a robot can be used to communicate and must act socially to transmit information effectively.

In all these areas, emotions play an important role in human behaviour, communication and interaction. Emotions are complex and are often closely related to the social context [32]. In recent years, facial expressions have been used more and more in this field, as we can see in papers such as [33,34,35,36,37]. In [33], the authors propose a system with three main steps: first an adaptive skin colour extraction, second the localization of the face and facial parts, such as eyes and mouth. Third, they propose to learn an objective function from training data. Experimental evaluation got a recognition rate of 70% using the Cohn–Kanade facial expression dataset, and 67% in a robot scenario. In [34] the authors combine a method for facial expression recognition based on active appearance models (AAMs) with eigen-faces dynamic face recognition. This method achieved a recognition rate of positive facial expressions (happy, surprise and anger) of about 85% and a recognition rate of negative facial expressions (disgust, sadness and fear) of about 65%. The authors did not implement the system in a social robot, but they proposed doing so as future work.

On the other hand, in [35] a novel approach to imitate facial expressions was presented, since imitating the facial expressions of another person is a significant signal within interpersonal communication. Another paper [36] presented an ethnographic study with 40 children from an elementary school. The participants interacted with a social robot, which was able to recognize and respond empathetically to some of the affective states of the children. The results suggested that the robot’s empathic behaviour affected children in a positive way. Recently, another study [37] proposed a model for adaptive emotion expression using the NAO robot. The NAO robot was able to express these emotions through its voice, posture, full-body postures, eye colour and gestures. The experiment was performed with 18 children and two NAO robots. One of the robots was an affective robot and the other a non-affective robot. The results showed that children react more expressively and more positively to an affective robot than to a robot that does not display emotions.

All the above mentioned studies demonstrate that facial expression recognition plays an important role in recognizing and understanding human expressions by robots. Many papers have studied facial expression recognition. There are several techniques on facial expression recognition, but recently deep learning methods have contributed to improving facial expression recognition, with works such as [17,18,19,20,21]. In [17] a model based on a single deep convolutional neural network (DNN) was proposed, which contained convolution layers and deep residual blocks. In [18] a combination of CNN and a specific image pre-processing step was proposed for the task of facial expression recognition. In [19] a hybrid convolution-recurrent neural network method was used. In [20] the performance of inception and VGG architectures, which are pre-trained for object recognition, were evaluated and these were compared with VGG-Face, which is pre-trained for face recognition. In [21] an ensemble of convolutional neural networks with probability-based fusion for facial expression recognition was presented, where the architecture of each CNN was adapted by using the convolutional rectified linear layer as the first layer and multiple hidden layers. Most of papers work with one or several datasets separately in order to improve current results [17,18,19,20,21]. That is training and testing sets belonging to the same dataset, but when we test with other databases different from the training set (a cross-dataset approach), the results can be very low [22,23,38]. In [22] the accuracy of the proposed deep neural network architecture in two different experiments—subject-independent and cross-dataset evaluation—were evaluated. In [23] the performance influence of fine-tuning CNN with a cross-dataset approach was investigated. In [38] a fine-tuned convolutional neuronal network for facial expression recognition and a specific image preprocessing method which is applicable to any facial expression dataset was proposed. The method was evaluated with five datasets, using both single and cross datasets protocols. Also, these datasets were combined for training purposes in order to obtain a more robust system under cross-dataset evaluation. The results improved significantly when the information captured with different cameras was merged. In order to verify the proper functionally of this CNN, it was compared with several CNNs [39,40,41,42] from the literature. The experiment consisted in using the same database and the same image pre-processing for all models. These models obtained 78.36%, 79.32%, 76.60% and 62.46%, respectively. The results showed that the proposed CNN (80.10%) is a competitive CNN with respect to other existing CNNs for facial expression recognition. The work finalized with a comparative experiment using both the proposed CNN and human assessment of 253 participants to recognize the facial expressions. The results showed that humans and machine are prone to similar misclassifications errors obtaining a difference of 14.63% between them. This is interesting since in human-robot interaction the robot needs to recognize the facial expression of any person, therefore the trained CNN must use a cross-dataset approach.

In the field of human-robot interaction, CNNs have been used in many papers [43,44,45,46,47,48]. In [43] a hybrid learning algorithm was proposed to study the reliability of the positioning accuracy of industrial robots more efficiently and accurately. In [44] an indoor scene classification method using a CNN to classify scenes with a novel feature matching algorithm was proposed.

Others papers such as [45,46,47] have used the CNN to recognize facial expressions using social robots. In [45] a CNN architecture based on emotions for robots was presented. The authors explained why it may be more effective to use a CNN compared to other methods to have better emotion in robots. In [46], an integration of a deep neural network (Mask R-CNN) with a mechanical robotic system is proposed. In this way, the system is more robust for human-robot interactive activities. In [47], the weight-adapted convolutional neural network (WACNN) is proposed to recognize basic facial expressions. The authors conducted an experiment using the proposed system on a social robot with seven volunteers. More recently, a similar work [48] to our paper proposed a novel deep convolutional neural network (CNN) architecture previously trained as a stacked convolutional automatic encoder (SCAE) for the recognition of emotions in unrestricted environments. It was evaluated in an uncontrolled environment using the NAO robot. Twenty-one men and seven women participated in the experiment. Finally, the authors asked three independent parties to label each collected image with the emotion they believed it represented. In this way, the authors could validate the images and overcome participant bias. Following this article, we test our own trained CNN using the same social robot (NAO) with more participants and validate the facial expression images with 10 experts instead of three as they suggest in [48].

The social robot NAO [13] has been used in many papers [14,15,16], where the social component of natural interaction is common to all the proposed applications, in addition to be a tool for motivation. In [14] a face detection method to track the faces of children with autism spectrum disorder in robotic assistive therapy was proposed. The intention of tracking the faces of autistic children is to measure the level of concentration of children in social interaction and communication using the humanoid robot NAO. In [15] the NAO robot for social care was evaluated in a smart home environment in short and long term. Eight elderly people tested a smart home robot system. The results showed that the participants trusted the little humanoid robot and that the participants were able to establish an emotional relationship with the robot. In [16] the challenges of playing with the NAO robot on a tablet were described. The authors chose the tic-tac-toe game and introduced interaction mechanisms to make it more enjoyable, with the goal of creating a template for the integration of HRI and machine learning.

The social robot NAO has proved to be a good choice for human-robot interaction. There are different types of questionnaires to measure the interaction [49,50]. In [49] human robot developers provided a simple set of tools to assess user acceptance of assistive social robots for elderly care settings. In [50] a questionnaire using social situations reported by a variety of people over six years was developed. From more than 10,000 collected situations, the “Social Interaction Questionnaire for Adults” (CISO-A) was constructed. The questionnaire was applied to 1573 subjects from various Spanish regions and with different careers. Both questionnaires [49,50] have the same interaction component, but they differ in the score. In [49] a score with values from 1 to 5 is used, while in [50] they use a score with values from 0 to 6. In our case, we decided to use a similar criterion to paper [49] but using a score with values from 1 to 4 to avoid a situation where a user gives a neutral response. In this way each user must decide, for example, if the interaction with the robot has been very good, good, bad or very bad.

For all the above mentioned reasons we present a system based on social robots, which can recognize the basic facial expressions and empathize with humans. Recently, a similar paper [48] proposed a novel deep convolutional neural network (CNN) architecture previously trained for the recognition of emotions in unrestricted environments. The difficulty of this work is that a CNN is able to recognize facial expressions on the wild, since the majority of works are trained and tested with the same databases [17,18,19,20,21]. Unlike [48], we test our own trained CNN using the same social robot (NAO) with more participants and we validate the facial expression images with 10 experts instead of three as suggested in [48]. We also measure the attention and interaction of the participants with a social robot through a questionnaire at the end of the experiment, in addition to study the difficulty to express a facial expression.

## 3. Experiment

The goal of this study is to measure both the interaction and the attention of users with the social robot NAO. In addition, we evaluate our trained neural network in real time with a completely new set of users. 

### 3.1. Design and Procedure

The first step was to guarantee an efficient interaction, without delays in the response and allowing a fluid natural communication. For this reason, part of the processing is done on a computer via Wi-Fi connection, since the CPU of the NAO robot is not very powerful. The NAOqi SDK is a software development kit, which manages and controls both the verbal communication and the movement of the engines of the NAO. In this application we used this software to create a fluid movement with the arms of the robot to simulate a gestural interaction and gain the user’s attention. These movements were performed synchronously when the robot was talking, to simulate a real dialogue. The frontal camera of the robot takes pictures with a resolution of 1280 × 960 pixels, to acquire images of the user, which are used to detect the face and recognize the facial expression.

#### 3.1.1. Image Pre-Processing and CNN

The images captured by the NAO robot are first analyzed by the method proposed in [51] to detect whether there is a face or not. If the face is detected, we get the eyes position using 68 facial landmarks proposed by [52]. From these landmarks, we calculate the geometric centroid of each eye and the distance between them. We draw a straight line in order to get the angle to rotate the image. The rotation of the axis that crosses the two eyes is then compensated and finally, the face is cropped. Finally, all images are converted to grayscale in range from 0 to 255 and resized to 150 × 150 pixels. This pre-processing step is important for a good recognition by the CNN, since this trained neural network uses this first pre-processing step in the training set.

Finally, the image is processed by the CNN (developed by the authors in [38]), to obtain the recognized expression. Since none of the participants of this experiment were included in any of the datasets (BU4FDE [53], CK+ (extended Cohn-Kanade) [54], Japanese Female Facial Expression (JAFFE) [55], Warsaw Set of Emotional Facial Expression Pictures (WSEFEP) [56] and Facial Expression, Gender and Age (FEGA) [38]) were the different datasets used to train the neural network used for training, and the results of this experiment can be considered as a test set that evaluates this CNN in a real environment.

#### 3.1.2. Application Design 

**Figure 1:**
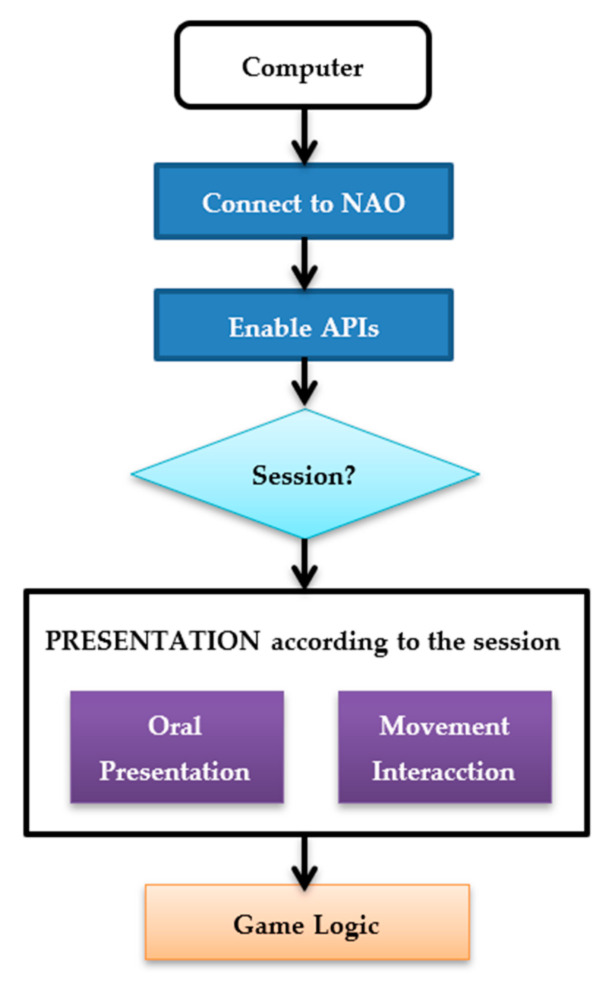
Game initializations. The interlocutor introduces the name of the user and selects the session in which the user will play.

**Figure 2:**
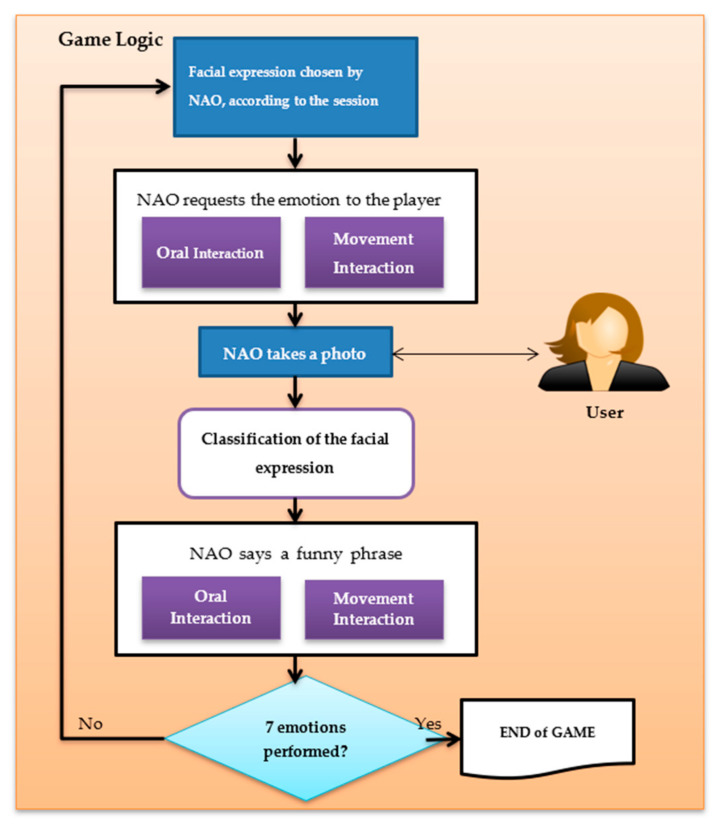
Game logic for both sessions.

In this subsection the structure of the game is explained (see Figure 1). First, a connection is established between the computer and the NAO robot. Second, the APIs responsible of speech and movement are enabled to allow it to initiate the interaction with the user. Third, the robot verifies the session in which is the game and varies its oral presentation according to the session, while making smooth movements with its arms in order to create a simulation of reality. In this presentation, the robot explains how the experiment will be performed by the user and the game logic begins (see Figure 2). This logic consists of selecting a facial expression from among seven facial expressions (anger, disgust, fear, happy, neutral, sad and surprise) according to the session initiated. Then, the robot begins to interact with the user, challenging the participant to show the proposed expression. The user performs the facial expression proposed by the robot and the robot takes a photo of the user. If the detection of the face is favourable, the image will be pre-processed and classified with the neural network. With this process, the robot is able to recognize the expression made by the user. For each recognized expression, the robot interacts with the user, trying to motivate and involve the user in the game through funny phrases. In case of not recognizing a face, the robot apologizes to the user and requests a replay of the facial expression. All this process is repeated until the seven facial expressions are performed, in order to finish the game correctly.

### 3.2. Experiment Design

**Figure 3:**
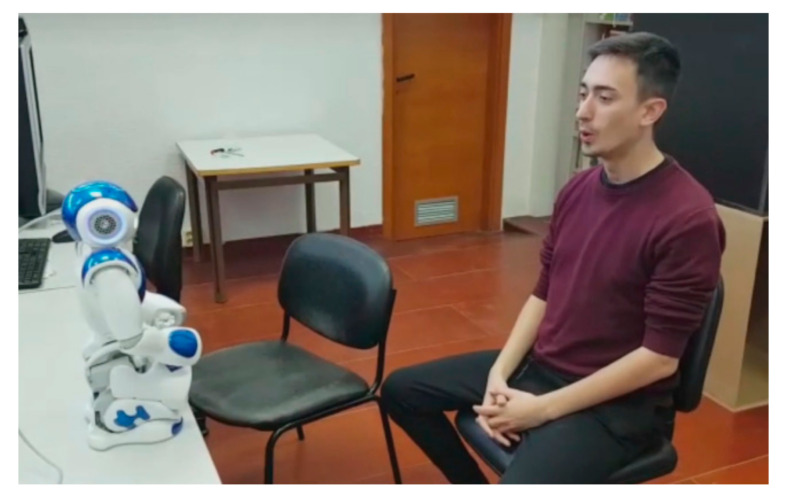
Interaction between the participant and the NAO robot. In this capture the robot recognizes the expression shown by the user.

**Figure 4:**
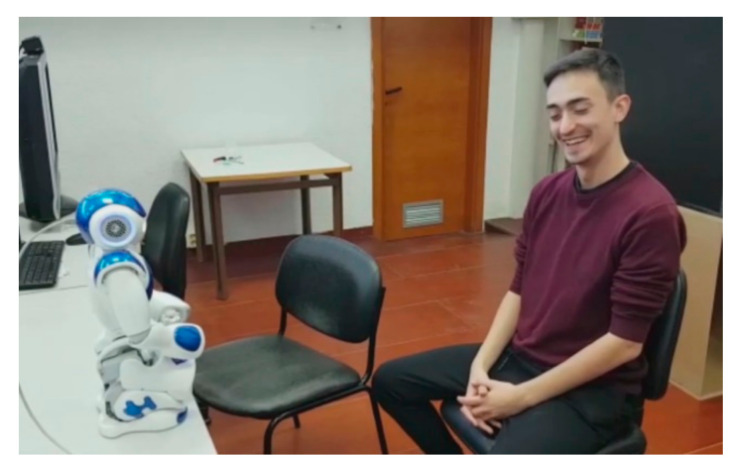
The reaction of the participant is shown in this figure. The robot’s answer with respect to the facial expression shown by the participant provoked a good reaction.

A total of 29 people participated in the experiment. Each participant was evaluated individually and signed the informed consent at the beginning of the experiment, since our robot would capture his or her images. The participant sat in front of the robot (see Figure 3) and followed the instructions of NAO, without any help from the interlocutor. The robot began with an explanatory presentation of the game and involved the user by addressing him or her by name, to give a sense of personalized application. In this presentation, the robot acts as if it were an evaluator of actors and actresses, challenging the participant to perform each one of the six basic expressions (happy, sadness, disgust, anger, surprise and fear) [57] in addition to the neutral expression. Each expression was evaluated with the CNN proposed in [38]. Then, the robot maintained a certain dialogue with the user depending on the recognized expression. These dialogs are usually funny phrases in relation to the expression made, and therefore, users usually smile and have a better user experience (see Figure 4). 

In Figure 3, we show the experiment with one of the participants. In this figure, we capture the moment when the user interpreted the expression of surprise. This facial expression was analyzed by the social robot to interact with the user. In Figure 4, we show a natural reaction of the participant when he heard the robot’s answer. Finally, the participants performed a questionnaire at the end of the experiment, where they evaluated this new experience in terms of interaction with the robot, attention in the game and difficulty of expression, among others questionnaire is available in the Appendix A).

#### 3.2.1. Participants

The experiment was performed with 29 participants between 18 and 38 years old, with an average age of 23.34 years. The 41% were women and 59% were men. The 97% of the participants did not have any previous experience with the NAO robot or any other social robot. The 79% of the participants considered themselves bad actors, compared to 21% who considered themselves good actors for this experiment.

#### 3.2.2. Sessions

Initially the number of sessions was not fixed. The number of sessions will be established when the users finished their learning by expressing emotions. It will end the sessions when users reach maximum expressiveness. Assuming it may take several sessions to feel comfortable with the interaction. Only two sessions were needed since the statistical analysis show that session 2 didn’t improve the session 1 results, so the experiment finished with the second session. Each session was launched in a personalized way with the name of the participant. The sessions have a length about 5 min using the social robot and about 10 min to respond the questionnaire. Each session had a number of interactions of seven interactions between the robot and participant. One for each expression performed. In the first session, the social robot introduced itself and gave the instructions to the user. The user had to carry out a sequence of expressions. This sequence consisted of perform the expressions from easiest to most difficult, with the neutral expression in the middle position. The expressions of happiness, surprise and anger were considered as the easiest expressions. The expressions of sadness, fear and disgust were considered as the most complicated to represent. In the second session the same exercise was performed but with a different presentation of the robot, much shorter, because the user already knew the game.

## 4. Results

In this section, we analyze the facial expression recognition results obtained both by the CNN and by 10 human experts, in addition to analyzing the results of the questionnaires which were completed by the users at the end of the experiments. Therefore, this section is divided into three parts. In the first part a comparison between the results obtained by the CNN and by the experts is done, in addition to an analysis of the difficulty to perform a particular expression by non-experts participants. Second, an analysis between two sessions has been performed. Finally, in the third part, the results of the questionnaires are analyzed.

To determine that the experts work on the same criteria, a study of inter-rater reliability is presented in Table 1. The inter-rate reliability has been computed using Cohen’s Kappa coefficient. Cohen’s Kappa between the same expert is always 1, and it is commutative. So, only has been presented coefficient between expert *a* and expert *b*, where *a < b*. If kappa = 1 implies maximum concordance, zero value means concordance produced by randomness, and negative values means discordance.

The Fleiss’ Kappa coefficient to have a statistical measure of inter-rate reliability between all experts has been computed. The value obtained is 0.7005, which indicates that there is a good concord, although it would be desirable for the value to be greater than 0.8. This reinforces the idea that it is difficult to determine a person’s facial expression.

### 4.1. Comparison between CNN and Human Experts

In this subsection, the results obtained by a trained CNN are analyzed together with the results obtained by 10 experts. One hundred and eighty-two images of the first session and 175 images of the second session were analyzed. If one of the sessions could not be performed due to user unavailability, no value is shown in the table (see Table 2, Table 3, Table 4 and Table 5). Because the neural network has been trained with five datasets (CK+, BU4DFE, JAFFE, WSEFEP and FEGA), d two of which do not contain the neutral face, we will show separately the results for both six and seven expressions. When analyzing the results obtained in the case of seven expressions, we shall take into account that the neutral face expression is under-represented in the training set.

#### 4.1.1. Results Using Six Expressions 

In Table 2, we show the results of each participant in the first session, obtained both by the CNN and by the experts (E1, E2, E3, E4, E5, E6, E7, E8, E9 and E10). In Table 3, we show the results of each participant in the second session. In both tables (Table 2 and Table 3), the six basic facial expressions are analyzed. As we see in both tables, the CNN achieves competitive results for six expressions (without neutral expression). 

One reason why the experts get better results is that the human capacity in facial expression recognition is more trained by the acquired experience of all their life. When they classified a facial expression and were not sure, they tried to remember what expression had not classified. 

The metric used in this paper is classification accuracy, the ratio of correct labelling (true positive—*TP*) to the total number of samples (*N*):

$$Accuracy=\frac{TP}{N}$$

Therefore, we tried to avoid this discarding by telling the experts that if they thought that two expressions were similar, they should label them with the same expression. In spite of this, the average classification accuracy obtained by our trained neural network is higher than for some experts in both sessions (1 and 2).

The best result in the first session is for expert E10, which obtained 12.6% more accuracy than the CNN. However, the best result in the second session is for expert E3, which obtained 10.1% more accuracy than the CNN. Nonetheless, the results obtained with our proposed CNN are competitive, with respect to other networks proposed in the literature using cross-datasets, since this experiment have allowed to collect a set of new images.

We have analyzed the CNN performance contrasting the expressions carried out by the users using a NAO social robot. To evaluate performance, we have compared CNN mean accuracy with ten experts’ mean accuracy. Accuracy has been taken as percentage, with values from 0 to 100. Comparison have been done for both sessions, in both sessions results show that CNN is far to achieve human performance.

We made a comparison between the accuracy of CNN and human. First a statistical model of human accuracy is computed, and subsequently the ranking of the CNN in this model is determined.

Experts mean accuracy have been modelled as a normal distribution. Shapiro-Wilk normality test is passed. Experts mean accuracy as a normal distribution is supposed as the null hypothesis, *p*-value computed are 0.7903 (W = 0.9604) for session 1, and 0.8725 (W = 0.9681) for session 2. So, we are able to model expertise accuracy as normal distribution with parameters N(70.035, 4.1383) for session 1, and N(69.468, 3.2142) for session 2.

**Figure 5:**
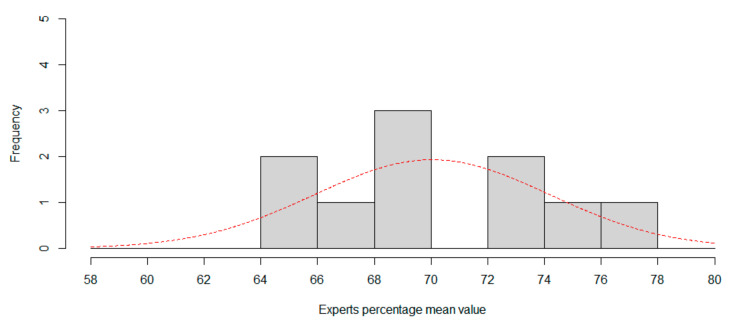
Session 1. Using six expressions, histogram of expertise mean value. Red line is best approximation for normal distribution.

**Figure 6:**
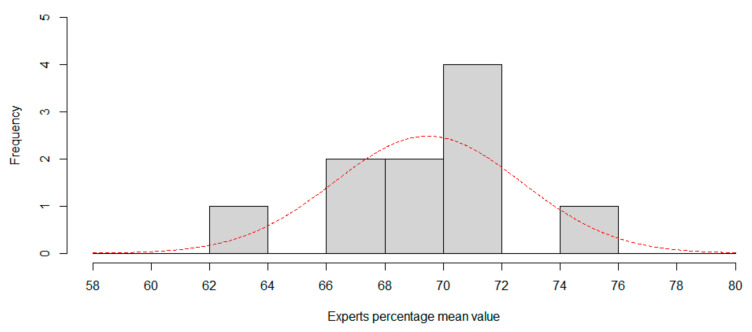
Session 2. Using six expressions, histogram of expertise mean value. Red line is best approximation for normal distribution.

Now, we can compute the CNN percentile in both distributions to rank CNN. For session1, percentile is 13.8694%, in that case one in 7.2 experts is worse than CNN. For session 2, the percentile is 6.8159%, so only one in 14.67 experts is worse than CNN, so CNN can be considered a low-accuracy expert classifier. Figure 5 and Figure 6 show the histogram for session 1 and session 2, using two percentage length bins. Best normal approximation is the plot in red color.

#### 4.1.2. Results Using Seven Expressions 

In Table 4 and Table 5, the eight facial expressions are analyzed. In Table 4, we show the results of each participant in the first session and in Table 5, we show their results in the second session, both by CNN and by experts. Both in Table 6 and Table 7, we show the results obtained for each facial expression by each expert and by the CNN trained with seven expressions. As we can see in both tables, the CNN trained with seven expressions obtain worse results than the experts. For this reason, we compare the results in detail (see Table 6 and Table 7). In Table 6 we show the results of session 1 and in Table 7, the results of session 2. The two last rows of these tables show the results between the average of the experts and the CNN. In these last rows of Table 6, we can observe that the CNN overcomes the experts in some facial expression such as happiness and anger, but surprise, sadness and disgust are better recognized by humans. Instead, fear is difficult to recognize both by humans and the CNN. The main difference in this first session is the neutral face. The experts recognize the neutral face with 68% more accuracy than the CNN. The CNN confused most of the neutral faces with angry faces. Nevertheless, the neutral face of women was recognized better by the CNN, although sometimes the neutral face was confused with an expression of anger or sadness. This problem can also be because in the training set of the trained CNN, there are fewer neutral faces because the CK+ and BU4DFE datasets do not contain the neutral face. And these two datasets are the largest of the five datasets used to train the CNN. In Table 7, we can see a similar situation to Table 6. In this case the CNN surpasses the experts in recognition of facial expressions such as surprise and anger, although, happiness, sadness and disgust are better recognized by humans. Like in Table 6, the main difference is in the neutral face, which is better recognized by experts. Although the CNN confuses the neutral face with the angry face, and this makes our average accuracy decrease about a 12% with respect to the experts. We can affirm that the CNN is mostly competitive, insomuch as this experiment is performed by non-expert participants in real time and it can be considered as a cross-validation experiment. Therefore, we can conclude that the CNN is close to the human perception, especially for the 6 basic expressions.

The same statistical analysis has been carried out for seven expressions, and the results conclude that CNN is not working fine adding neutral expression. Again, experts mean accuracy as a normal distribution is supposed as the null hypothesis, *p*-value computed are 0.4418 (W = 0.9294) and 0.6767 (W = 0.9507) for session 1 and session 2. Experts mean accuracy are modelled as normal distribution with parameters N(71.426, 3.8986) for session 1, and N(71.284, 3.5900) for session 2. Now, we can compute the CNN percentile in both distributions to rank CNN. For session1, percentile is 0.04490579%, in that case one in 2227 experts is worse than CNN. For session 2, the percentile is 0.02986%, so only one in 3349 experts is worse than CNN.

**Figure 7:**
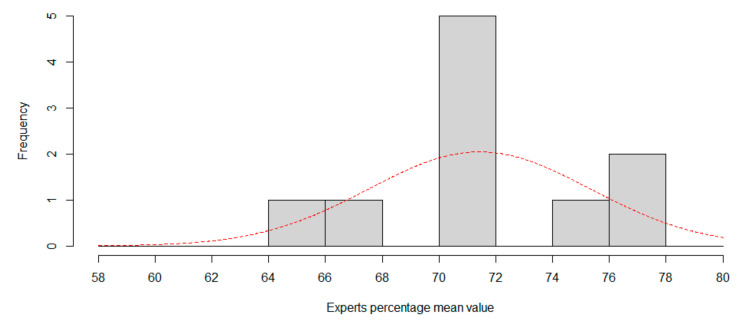
Session 1. Using seven expressions, histogram of expertise mean value. Red line is best approximation for normal distribution.

**Figure 8:**
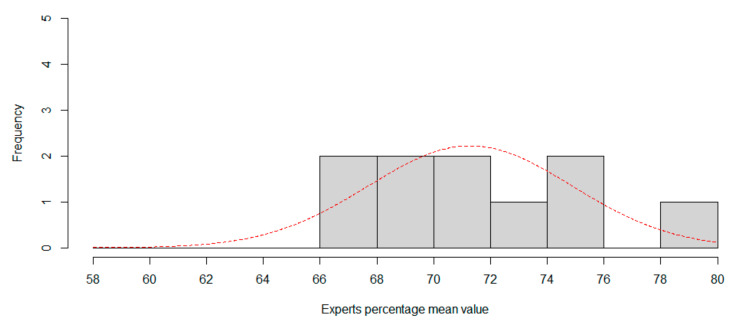
Session 2. Using seven expressions, histogram of expertise mean value. Red line is best approximation for normal distribution.

This experiment portends that while for humans the neutral expression is easily recognizable, for the CNN it is a problem, presumably since the six expressions previously evaluated are at the midpoint. Figure 7 and Figure 8 show the histogram for session 1 and session 2, using two percentage length bins. Best normal approximation is plot in red color.

#### 4.1.3. Difficulty of Expression Recognition for Users and Experts 

Another question that arose during the experiment was the difficulty, for each participant, of representing the different facial expressions, since most of them doubted in some expression. This caused the bad capture of some images. For this reason, we needed experts to evaluate the images, so we compare and verify the results. In addition to evaluate the images by experts, we measured this difficulty of the participants to express themselves through a questionnaire, where they rated between 1 and 4 (1 the least and 4 the most difficult) the difficulty to represent each of the facial expressions (see Table 8).

In Table 8, we display the mean recognition accuracies of the two sessions obtained both by experts and the CNN and compared them with the average difficulty ratings in interpreting each facial expression. We observe that the facial expressions more difficult to express by the participants are disgust, sadness and fear, which obtain a score equal or greater than 2. These results correlate with the recognition accuracy results obtained by the CNN, which are the lowest. The easiest facial expressions to interpret, according to the participants, are angry, happy, neutral and surprise, which obtain a score lower than 2. These results match with better recognition accuracies in both cases (CNN and experts), except in the angry face expression in the case of the human experts, because, in case of doubt they always chose the neutral expression. Pearson correlation coefficients has been calculated. Correlation between “Difficulty to express facial expressions” and “Expert’s accuracy” is −0.8207. This correlation is interpreted as meaning that the expressions that are most difficult to perform are also the expressions that are least easily recognized by experts. On the other hand, correlation between “Difficulty to express facial expressions” and “CNN accuracy” is –0.5506. This implies that the correlation is lower or not linear, probably since the CNN accuracy is lower. Removing the neutral expression, Pearson correlation coefficient between “Difficulty to express facial expressions” and “CNN accuracy” is –0.9342. Then, it can be determined that there is a strong linear correlation, but the neutral expression is not well recognized by the CNN.

**Figure 9:**
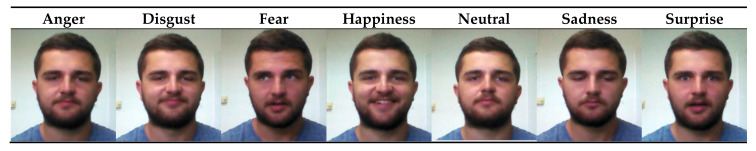
Interpretation of the seven expressions (49% recognized by experts, 43% recognized by CNN).

**Figure 10:**
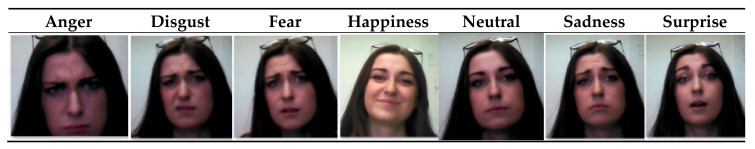
Interpretation of the seven expressions (94% recognized by experts, 100% recognized by CNN).

This explains why the results of the neutral face in the evaluation by experts were high. In Figure 9 and Figure 10, we show two extreme cases in the representation of facial expressions by the participants. In Figure 10 the experts recognized a mean of 94% of their expressions (seven experts recognized 100% of the expressions, two experts recognized 86% of them and one expert recognized 71% of them). The CNN recognized all of them (100%), coinciding with the majority of experts. In Figure 9 the experts only recognize a mean of 49% of the expressions (6 experts recognized 43% of them and 4 experts recognized only 57% of them). Finally, the CNN recognized 43% of the expressions, coinciding with the majority of experts. In both figures the order of the expressions is the same. As we can see, performing this type of experiment with non-actor participants leads to interpretation difficulties both for neural networks and humans.

### 4.2. Comparison between Two Sessions

This subsection discusses the differences between the two sessions. We reach the conclusion that there are no significant differences between the classification results of the experts between both sessions. This verifies that users perform expressions correctly from the first session. The data used for the analysis is shown in Table 9, and users who have only attended one of the two sessions have been omitted as they are paired data.

A paired difference test is used to contrast the mean difference from two sessions. To determine the most related test, first we analyze the normality of the differences. Shapiro-Wilk normality test has been computed. Null hypothesis is the difference between sessions is a normal distribution. Process results is *p*-value = 0.1364 (W = 0.9322), so we assume a normal distribution for difference.

To demonstrate that two sessions are equal, we demonstrate that there are no significant differences. Due to normality distribution of difference we apply a paired samples t-test to compare the means accuracy between two sessions. Null hypothesis is mean value of the differences is 0 (no difference between sessions). The *p*-value is 0.7333 (t = 0.34536 and df = 21), so we conclude that two sessions are statistically identical, and there is no worsening or improvement between the two sessions.

### 4.3. Results of the Questionnaire 

**Figure 11:**
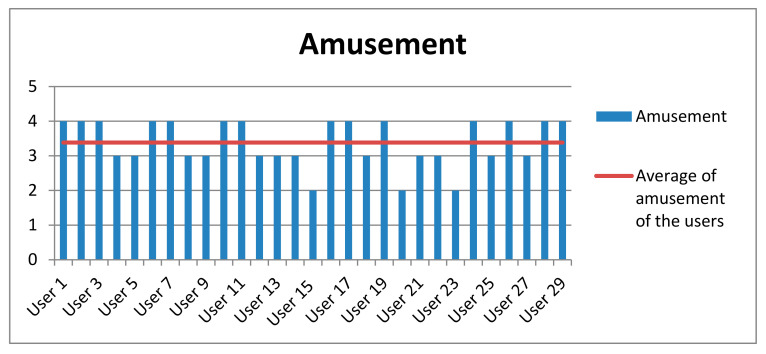
Results obtained in the questionnaire performed by the participants about the amusement obtained with the social robot.

**Figure 12:**
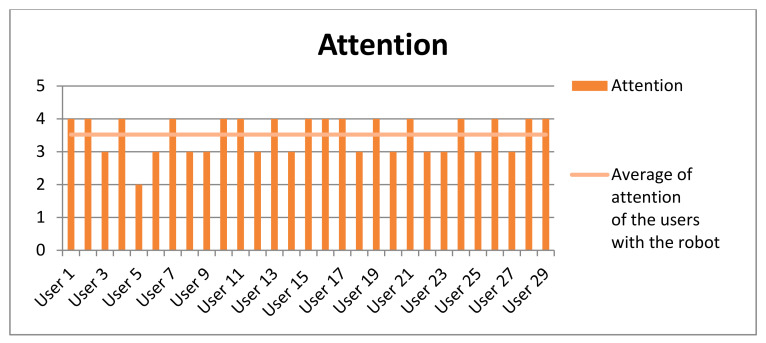
Results obtained in the questionnaire performed by the participants about the attention obtained with the social robot.

**Figure 13:**
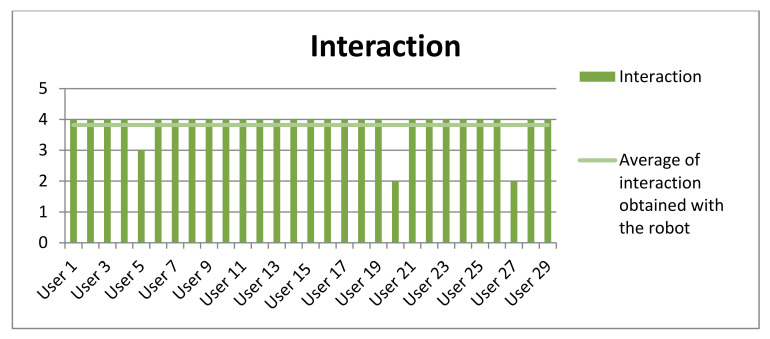
Results obtained in the questionnaire performed by the participants about the interaction obtained with the social robot.

Finally, participants were surveyed at the end of the experiment. Most of the users (93%) did not need any help. They were guided only by the robot’s instructions. Only 7% of the participants asked the interlocutor some questions. Table 9 shows the averages obtained both in the level of amusement and interaction experienced by the participants as well as their level of attention in the game. This measure was evaluated between 1 and 4 (1 for the lowest and 4 for the highest). These high results show that the participants of this experiment obtained a quite satisfactory experience (see Figure 11, Figure 12 and Figure 13).

Among the comments left by the participants, we highlight that they liked the experience of being able to interact with a social robot, that the robot was able to recognize their facial expressions and be able to evaluate their capacity as an actor or actress. The funny dialogues that the robot had according to the recognized expression and the harmonious movements that the NAO robot performed when interacting with the user, resulted in a satisfactory user experience.

## 5. Conclusions

We have designed, implemented and validated a multimodal interaction system based on a social robot which allows to evaluate a trained neural network in facial expressions which permit to test the CNN in a real environment with a completely new set of users. Also, the system can measure the attention and interaction of the participants with a social robot through a questionnaire at the end of the experiment. We also demonstrated that our proposed method offers state-of-the-art classification performance on unseen data collected in uncontrolled environments with a Nao robot.

In this way, an important novelty in HCI context is provided, since the social robot leads the process of capturing expressions through gestural, visual and auditory interaction. The social robot establishes a script in the human-social robot interaction process, and therefore, enhances an empathic relationship between both.

For this, a convolutional neural network (CNN) has been used in the application of the social robot. This system has been validated in 29 non-expert users. We have shown that the CNN is mostly competitive, taking into account that this experiment is performed by non-expert participants in real time and can be considered a cross-validation experiment.

According to the results, the social robot can be used as a tool in the interaction with people to learn basic expressions, so it can be used as a tool for training/learning facial expressions, where the social robot acts as a supervisor of the user’s level of success regarding the expression performed. This system allows replicating and learning in a playful way six facial expressions (happiness, sadness, fear, surprise, angry and disgust) and the neutral expression. The results show that the CNN is close to human perception, especially for the six basic expressions. However, the CNN fails in the neutral expression recognition. The most plausible cause is that neutral expression doesn’t appear in different datasets. Therefore, the CNN confused most of the neutral faces with angry faces. In future work, we will try to resolve this question applying more information in the training set or improving the pre-processing step in order to recognize better this kind of image. On the other hand, a study has been performed in order to determine the level of fun, interaction and attention that the participants experienced in the game. These results show that the participants of this experiment obtained a quite satisfactory experience. In our opinion empathy is a key part in human-human communication, so replication of such for social robots is important. The development of real time expression/emotion recognition will aid in the creation of empathetic robots and possibly an increase in acceptance of robots in society. This work is a preliminary study to design more complex emotions recognition models that use facial expressions, contextual cues and bio-signals (heart rate, electrodermal conductivity and EEG). 

As future work, it is planned to perform this same experiment with several sessions, especially for children with attention deficit disorder. We also plan to explore a generative adversarial network as these have been demonstrated to produce remarkable results for expression recognition.

## Figures and Tables

**Table 1:** Inter-rate reliability between pair of experts. Cohen’s Kappa coefficient has been computed, minimum kappa value is 0.6157 between experts 2 and 7, maximum kappa value is 0.7851 between experts 4 and 10.

Expert	2	3	4	5	6	7	8	9	10
1	0.6745	0.7367	0.7187	0.7007	0.6828	0.6457	0.6851	0.7192	0.6941
2		0.6795	0.6966	0.6777	0.6704	**0.6157**	0.6405	0.6572	0.6866
3			0.7524	0.7480	0.6974	0.6497	0.6894	0.6970	0.7426
4				0.7359	0.7693	0.6961	0.7351	0.7658	**0.7851**
5					0.7522	0.6565	0.7053	0.7176	0.7405
6						0.6484	0.6815	0.7080	0.7299
7							0.6850	0.6524	0.6902
8								0.7012	0.6885
9									0.7452

**Table 2:** Comparison between CNN and human experts for the six basic facial expressions. An empty value in the table corresponds to users who could not perform session 1 and only performed session 2. We show the average of the results obtained by the CNN and by the best and worst experts in bold text.

Participants (Session 1)	CNN	E1	E2	E3	E4	E5	E6	E7	E8	E9	E10
User 1	83%	100	83	100	83	100	67	67	100	50	67
User 2	83%	83	67	100	83	83	83	83	83	83	83
User 3	67%	67	100	33	100	100	100	100	67	67	67
User 4	40%	60	40	60	40	60	60	60	60	60	60
User 5	67%	67	67	83	83	67	67	67	83	67	67
User 6	75%	50	50	100	50	50	50	50	50	50	50
User 7	83%	83	83	83	83	83	83	67	83	83	83
User 8	50%	67	50	67	50	50	67	33	33	67	67
User 9	83%	83	83	83	83	67	83	100	83	67	83
User 10	--	--	--	--	--	--	--	--	--	--	--
User 11	75%	100	100	100	100	100	75	100	100	100	100
User 12	33%	67	67	67	67	67	67	33	50	67	33
User 13	60%	100	80	80	100	60	10	60	100	80	100
User 14	60%	60	60	60	60	60	80	60	60	20	60
User 15	83%	67	83	83	67	67	83	50	50	67	67
User 16	50%	67	67	83	83	67	83	33	83	67	67
User 17	75%	25	25	50	50	50	50	25	25	25	100
User 18	60%	20	40	40	40	40	60	80	60	40	60
User 19	33%	67	83	33	67	83	50	50	67	67	67
User 20	--	--	--	--	--	--	--	--	--	--	--
User 21	50%	83	83	83	83	83	83	83	83	100	100
User 22	80%	50	0	75	50	0	75	50	75	50	50
User 23	67%	67	50	33	50	50	50	33	33	50	83
User 24	67%	80	80	100	100	80	80	100	80	80	100
User 25	67%	83	67	83	100	83	67	83	83	100	100
User 26	60%	75	75	75	100	100	100	75	100	100	100
User 27	60%	40	40	60	80	40	40	40	20	40	80
User 28	67%	83	83	67	100	83	100	83	83	100	100
User 29	--	--	--	--	--	--	--	--	--	--	--
**Average**	**64.6%**	69	65.6	72.4	75.1	68.2	73.2	**64.1**	69.1	67.1	**76.7**

**Table 3:** Comparison between CNN and human experts for the six basic facial expressions. An empty value in the table corresponds to users who could not perform session 2 and only performed session 1. We show the average of the results obtained by CNN and by the best and worst experts in bold text.

Participants (Session 2)	CNN	E1	E2	E3	E4	E5	E6	E7	E8	E9	E10
User 1	83%	83	67	100	83	83	83	83	83	83	67
User 2	67%	67	83	100	67	83	83	33	67	67	67
User 3	100%	100	100	100	83	83	100	100	67	100	100
User 4	50%	50	67	33	50	50	50	33	50	50	33
User 5	67%	67	67	67	67	67	67	50	67	83	67
User 6	67%	67	67	83	83	100	100	100	83	83	100
User 7	67%	83	83	100	83	100	100	83	83	83	100
User 8	67%	33	33	33	33	33	33	33	33	33	33
User 9	67%	67	50	83	67	50	67	50	50	67	50
User 10	50%	50	50	67	50	33	50	67	67	17	83
User 11	--	--	--	--	--	--	--	--	--	--	--
User 12	67%	80	60	60	80	80	60	80	80	60	60
User 13	50%	100	100	83	67	83	100	100	67	67	100
User 14	--	--	--	--	--	--	--	--	--	--	--
User 15	67%	33	50	50	50	67	50	50	50	33	50
User 16	33%	83	83	83	100	67	83	33	67	83	100
User 17	--	--	--	--	--	--	--	--	--	--	--
User 18	--	--	--	--	--	--	--	--	--	--	--
User 19	75%	100	100	100	100	100	75	100	100	75	100
User 20	60%	17	33	33	17	17	17	17	33	17	17
User 21	67%	67	67	67	67	67	67	67	67	83	67
User 22	50%	60	100	80	120	100	60	100	100	100	100
User 23	67%	67	67	67	83	83	67	67	67	67	83
User 24	50%	67	50	67	67	67	50	17	33	50	67
User 25	83%	100	100	100	83	100	100	100	83	83	100
User 26	83%	83	83	83	83	83	83	83	83	83	83
User 27	33%	33	33	67	33	33	33	33	33	33	33
User 28	67%	83	83	83	67	67	83	33	83	67	67
User 29	80%	80	80	100	80	80	80	80	80	100	60
**Average**	**65.5%**	68.8	70.3	**75.6**	70.5	71.1	69.7	**63.7**	67.1	66.7	71.5

**Table 4:** Comparison between CNN and human experts for the seven facial expressions. An empty value in the table corresponds to users who could not perform session 1 and only performed session 2. We show the average of the results obtained by CNN and by the best and worst experts in bold text.

Participants (Session1)	CNN	E1	E2	E3	E4	E5	E6	E7	E8	E9	E10
User 1	71%	100	86	100	86	100	71	71	100	57	71
User 2	86%	86	71	100	86	86	86	86	86	86	86
User 3	75%	75	100	50	100	100	100	100	75	75	75
User 4	33%	67	50	67	50	67	50	67	67	67	50
User 5	67%	67	67	83	83	67	67	67	83	67	67
User 6	75%	50	50	100	50	50	50	50	50	50	50
User 7	83%	83	83	83	83	83	83	67	83	83	83
User 8	43%	71	43	71	57	57	57	29	43	71	71
User 9	71%	86	86	86	86	71	71	100	86	71	86
User 10	--	--	--	--	--	--	--	--	--	--	--
User 11	60%	80	100	100	80	100	60	80	80	100	100
User 12	43%	71	57	71	71	71	57	43	57	71	43
User 13	50%	100	83	83	100	50	100	50	83	83	83
User 14	50%	67	50	67	67	67	83	50	67	33	67
User 15	71%	71	86	86	71	71	86	57	57	71	71
User 16	43%	71	71	86	86	71	86	43	86	71	71
User 17	60%	40	40	60	60	60	60	40	40	40	100
User 18	50%	17	33	50	50	50	67	67	67	50	67
User 19	33%	67	83	33	67	83	50	50	67	67	67
User 20	--	--	--	--	--	--	--	--	--	--	--
User 21	43%	71	71	86	86	86	86	71	86	100	100
User 22	67%	60	20	60	60	0	80	60	80	60	60
User 23	57%	71	57	43	57	57	57	43	43	57	86
User 24	57%	83	83	100	100	83	67	100	83	83	100
User 25	57%	86	71	86	100	86	71	86	86	100	100
User 26	67%	80	80	80	100	100	100	80	100	100	100
User 27	60%	40	40	60	80	40	40	40	20	40	80
User 28	57%	86	86	71	100	86	100	86	86	100	100
User 29	--	--	--	--	--	--	--	--	--	--	--
**Average**	**58.9%**	71.0	**67.3**	75.5	77.5	70.9	72.5	64.7	71.5	71.4	**78.2**

**Table 5:** Comparison between CNN and human experts for the seven facial expressions. An empty value in the table corresponds to users who could not perform session 2 and only performed session 1. We show the average of the results obtained by CNN and by the best and worst experts in bold text.

Participants (Session 2)	CNN	E1	E2	E3	E4	E5	E6	E7	E8	E9	E10
User 1	71%	100	71	100	86	86	86	86	86	86	71
User 2	57%	57	86	100	71	86	71	43	71	71	71
User 3	100%	100	100	100	86	86	100	100	71	100	100
User 4	43%	43	57	43	57	57	43	43	43	57	43
User 5	57%	100	57	71	71	71	71	57	71	86	71
User 6	57%	86	71	86	86	100	100	86	71	86	100
User 7	67%	100	71	100	86	100	86	86	86	86	100
User 8	67%	33	33	33	33	33	33	33	33	33	33
User 9	57%	17	67	100	83	67	83	67	67	83	67
User 10	43%	57	43	71	57	43	57	71	71	29	86
User 11	--	--	--	--	--	--	--	--	--	--	--
User 12	67%	100	50	67	83	83	67	67	67	67	67
User 13	43%	86	86	86	71	86	100	100	71	71	100
User 14	--	--	--	--	--	--	--	--	--	--	--
User 15	71%	57	57	57	57	71	57	57	57	43	57
User 16	29%	57	86	86	100	71	86	43	71	86	100
User 17	--	--	--	--	--	--	--	--	--	--	--
User 18	--	--	--	--	--	--	--	--	--	--	--
User 19	60%	80	80	80	80	80	60	80	80	60	80
User 20	50%	50	33	50	33	33	33	33	50	33	33
User 21	57%	71	57	71	71	71	71	71	71	86	71
User 22	50%	67	100	67	100	100	67	100	100	100	100
User 23	57%	57	57	71	86	86	71	71	71	71	86
User 24	57%	71	57	71	71	71	57	29	43	57	71
User 25	71%	100	86	100	86	100	100	100	86	86	100
User 26	86%	86	86	86	86	86	86	86	86	86	86
User 27	33%	50	43	71	43	50	50	50	50	43	43
User 28	57%	43	71	86	71	71	71	43	86	71	71
User 29	67%	67	67	100	67	67	67	67	67	83	50
**Average**	**59.0%**	69.4	66.9	**78.2**	73.6	74.2	71.0	**66.7**	69.1	70.4	74.3

**Table 6:** Accuracy rate of each facial expression, in the first session, by the 10 experts and by the CNN, in addition to their mean. In two last files we show the main differences.

Session 1	Anger	Disgust	Fear	Happiness	Neutral	Sadness	Surprise	Mean
E1	76%	95	24	88	86	58	76	71.0
E2	72%	79	14	92	76	54	80	67.3
E3	68%	84	33	92	95	71	84	75.5
E4	72%	84	33	92	95	75	88	77.5
E5	64%	79	24	83	90	79	76	70.9
E6	72%	74	33	88	71	83	80	72.5
E7	48%	68	19	96	71	54	88	64.7
E8	72%	74	38	88	90	67	72	71.5
E9	60%	89	29	88	100	63	76	71.4
E10	76%	79	43	92	90	79	84	78.2
Mean Experts	68%	81	29	90	87	68	80	72
CNN	76%	48	29	100	19	50	72	58.9

**Table 7:** Accuracy rate of each facial expression, in the second session, by the 10 experts and by the CNN, in addition to their mean. In two last files we show the main differences.

Session 2	Anger	Disgust	Fear	Happiness	Neutral	Sadness	Surprise	Mean
E1	63%	63	25	92	90	76	83	69.4
E2	63%	79	13	100	81	88	48	66.9
E3	79%	79	29	92	95	88	91	78.2
E4	50%	83	25	100	81	84	96	73.6
E5	54%	75	38	96	86	80	96	74.2
E6	46%	71	46	100	76	84	78	71.0
E7	50%	58	29	96	71	76	87	66.7
E8	46%	75	21	96	100	68	83	69.1
E9	46%	88	29	88	86	68	96	70.4
E10	54%	75	33	96	90	84	96	74.3
Mean Experts	55%	75	29	96	86	80	85	71.3
CNN	63%	63	25	92	14	56	92	59

**Table 8:** Comparison between the mean of experts, CNN and the opinions of the participants about the difficulty to express facial expressions. These means were calculated from two sessions performed for each facial expression.

Difficulty to Express Facial Expressions	Anger	Disgust	Fear	Happiness	Neutral	Sadness	Surprise
Mean Participants	1.34	2.07	3.07	1.10	1.45	2.00	1.69
Mean recognition accuracy Experts	62%	78%	29%	93%	86%	74%	83%
Mean recognition accuracy CNN	70%	56%	27%	96%	17%	53%	82%

**Table 9:** Results obtained by experts in both sessions. Data used to contrast users carry out expressions in first session. Mean accuracy value retrogress in second session.

Participants	Session 1 by 10 Experts (Mean)	Session 2 by 10 Experts (Mean)
User 1	84%	86%
User 2	86%	73%
User 3	85%	94%
User 4	60%	49%
User 5	72%	73%
User 6	55%	87%
User 7	82%	90%
User 8	57%	33%
User 9	83%	70%
User 12	61%	72%
User 13	82%	86%
User 15	73%	57%
User 16	74%	79%
User 19	63%	76%
User 21	84%	71%
User 22	54%	90%
User 23	57%	73%
User 24	88%	27%
User 25	87%	94%
User 26	92%	86%
User 27	48%	49%
User 28	90%	69%
**Average**	**72.0%**	**70.1%**

## Appendix A

In this section the questionnaire used in this work is shown.
